# Assessing the Impact of Lifestyle Interventions on Diabetes Prevention in China: A Modeling Approach

**DOI:** 10.3390/ijerph16101677

**Published:** 2019-05-14

**Authors:** Linna Luo, Bowen Pang, Jian Chen, Yan Li, Xiaolei Xie

**Affiliations:** 1 School of Economics and Management, Tsinghua University, Beijing 100084, China; luoln.13@sem.tsinghua.edu.cn (L.L.); chenj@sem.tsinghua.edu.cn (J.C.); 2 Research Center for Contemporary Management, Key Research Institute of Humanities and Social Sciences at Universities, Tsinghua University, Beijing 100084, China; 3 Center for Healthcare Service Research, Department of Industrial Engineering, Tsinghua University, Beijing 100084, China; pzkaixin@foxmail.com; 4 Center for Health Innovation, The New York Academy of Medicine, New York, NY 10029, USA; yli@nyam.org; 5 Department of Population Health Science and Policy, Icahn School of Medicine at Mount Sinai, New York, NY 10029-5674, USA

**Keywords:** agent-based modeling, non-communicable disease, diabetes epidemic, social influence, lifestyle interventions

## Abstract

China’s diabetes epidemic is getting worse. People with diabetes in China usually have a lower body weight and a different lifestyle profile compared to their counterparts in the United States (US). More and more evidence show that certain lifestyles can possibly be spread from person to person, leading some to propose considering social influence when establishing preventive policies. This study developed an innovative agent-based model of the diabetes epidemic for the Chinese population. Based on the risk factors and related complications of diabetes, the model captured individual health progression, quantitatively described the peer influence of certain lifestyles, and projected population health outcomes over a specific time period. We simulated several hypothetical interventions (i.e., improving diet, controlling smoking, improving physical activity) and assessed their impact on diabetes rates. We validated the model by comparing simulation results with external datasets. Our results showed that improving physical activity could result in the most significant decrease in diabetes prevalence compared to improving diet and controlling smoking. Our model can be used to inform policymakers on how the diabetes epidemic develops and help them compare different diabetes prevention programs in practice.

## 1. Background

There are 415 million people with diabetes in the world. A total of 642 million people is expected to develop diabetes by 2040 [1]. As a prevalent non-communicable metabolic disease, diabetes can lead to many serious complications such as blindness and lower-extremity amputation [2,3]. Global aging and the growing trend of obesity and unhealthy lifestyles are major contributors to the diabetes epidemic [4,5,6]. Previous studies have shown that lifestyle modifications may be a more effective in preventing and controlling diabetes compared to medical interventions for people with pre- and early-stage diabetes [7,8]. Moreover, the trials implemented in China showed that diet and exercise brought a significant reduction in the diabetes rate of the trial population over a six-year period [9].

Social influence plays an important role on people’s lifestyle; behavioral changes are more likely to happen when individuals receive social reinforcement from multiple neighbors in their social networks [10]. People can be influenced by their social network to adopt or change behaviors which will affect their health in the future [11]. Social influence also has an impact on adolescents’ lifestyle and attitudes [12]. Specifically, certain lifestyles such as excessive consumption of unhealthy food may spread between different social relations (friends, siblings) [13]. The spread of smoking behavior and smoking cessation turn out to be relevant to the network phenomenon [14]. Exercise habits have been shown to be influenced by social relations [15].

The Framingham Heart Study quantitatively analyzed the peer influence of obesity. They recorded the weight changes of each individual in a highly dense interconnected social network. They found that social contacts influenced people’s behavior and determined their risk of becoming obese [16]. Social influence may be one of the key factors for understanding the epidemic of non-communicable disease [17]. Since social influence can modify behavior changes through social networks [18], mathematical modeling of social influence and quantitative assessments is needed to help policy makers to compare and choose different intervention programs. 

Markov models and system dynamic models have been developed to study the progression and complications of diabetes [19,20]. As the population ages, the burden of chronic disease becomes a great challenge to the healthcare system. Agent-based modeling was proposed as a promising approach to study public health outcomes and investigate complex public health problems, since, it can capture the feedback between individual-level behaviors and population-level characteristics [17]. Systematic reviews [21,22] summarized the recent agent-based models of non-communicable chronic disease, and several agent-based models have already been developed to assist diabetic retinopathy research; however, more is needed to help improve population health management. A number of agent-based models are well designed to explore the contagion of certain behavioral habits and compare the effects of different interventions and policies for improving people’s living habits (i.e., eating healthier, exercising more) [23,24]. 

Diabetes is caused by the interplay of individual conditions (i.e., lifestyle, family history) and the external environment, and its complications may lead to serious health outcomes, even death. Given the serious health and economic burden brought by the diabetes epidemic, lifestyle interventions are more favorable compared to metformin treatment [19]. The diabetes epidemic is a complex problem, and the feedback mechanism of how individual diabetes-related behaviors influence population-level diabetes prevalence is worth exploring. This study was intended to gain insight into this feedback mechanism. We developed an agent-based model of diabetes based on the risk factors and related complications of diabetes to inform decision makers on how the diabetes epidemic develops in China. Our model generated a user-specific population, captured the progression of individual health conditions, and reported the population health outcome. Moreover, we described the person-to-person interaction and peer influence of certain lifestyles quantitatively by using simple rules of social interaction. To the best of our knowledge, this is the first agent-based model of diabetes that has incorporated social interaction. Our analysis contained two key factors which led to the rising incidence of diabetes: (1) The individual-level diabetes health progression, (2) the social interactions which influence the individual diabetes-related behavior that then determines the population-level characteristics. We also calibrated and validated our model with Chinese data. We used the model to assess the long-term impact of a set of hypothetical lifestyle interventions on the diabetes rate. Our model may serve as a decision support tool for public health policy makers to predict the effectiveness of different lifestyle interventions without spending resources comparing different programs in the field.

## 2. Study Area 

The Western Pacific Region has 153 million adults with diabetes; over 63% of adults in this region live in China. China has the largest number of people with diabetes in the world, and its diabetes epidemic is getting worse. A total of 1.3 million deaths occurred in China due to diabetes in 2015, with 40.8% of those deaths occurring in people under 60. According to the National Health and Family Planning Commission, Chinese people are developing diabetes at a lower body weight than Americans; poor nutrition in early life and over nutrition in old agelater life contribute to the diabetes epidemic in China.

The continually rising diabetes rate places a heavy burden on the health system, and; also indicates the future epidemic of diabetes-related complications (i.e., retinopathy, neuropathy, cardiovascular disease). The health system is facing a major challenge due to the diabetes problem. Furthermore, China is a developing country in East Asia; the cost of treating diabetes and diabetic complications incurs a huge financial burden on the economic system. China features a dense population which lead to a highly interconnected social network system; people interact with each other and form their own lifestyle which affects their risk of diabetes. Therefore, it is reasonable and necessary to take social influence into consideration when studying this epidemic’s phenomenon in China.

## 3. Methods 

Agent-based modeling is a bottom-up approach which allows us to capture the progression of individual-level health and social interactions in a population at the same time. We defined agents and generated a user-specific population; we simulated the individual diabetes-related behaviors and health progressions and then observed the population health outcome (i.e., diabetes rate). Our model aimed to inform policy makers on how the diabetes epidemic develops and help them compare different diabetes prevention programs in practice.

### 3.1. The Individual Health Progression 

Each agent was a basic unit and endowed with some characteristics: Two demographic factors (age and gender), three behavioral factors (smoking, physical activity, and diet), and several diabetes-related health factors (body weight, cholesterol, blood pressure, blood glucose, diabetic retinopathy, nephropathy, and cardiovascular disease). These factors were selected to capture the symptoms, causes, and complications of diabetes [25,26]. Among all the factors, age and gender are intrinsic factors which are not affected by other factors; gender is always the same, and a single time period is defined as one year, so people’s age incrementally increased by one pereach time period of during the simulation. The other factors changed interactively during the simulation.

**Figure 1:**
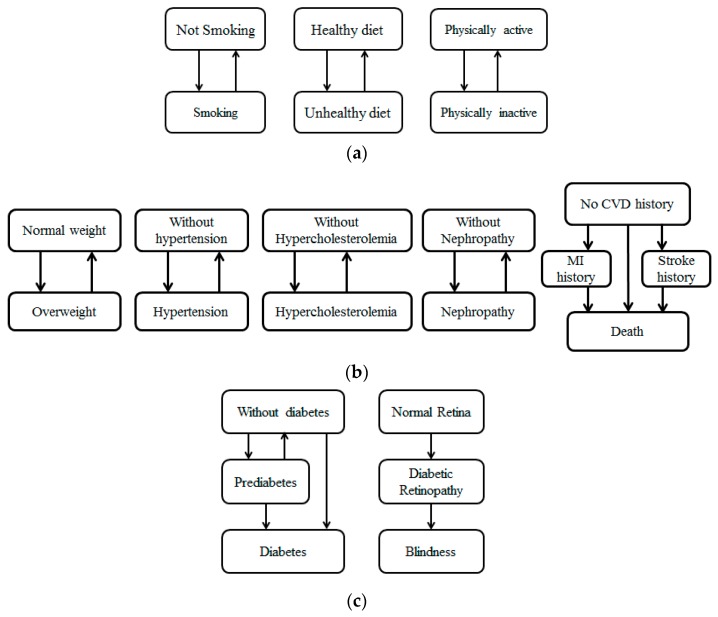
Individual health progression: (**a**) Health behavior state; (**b**) diabetic complication state; (**c**) diabetes-related health outcome state.

As Figure 1 shows, we developed eleven state charts to capture the changes in behaviors and diabetes-related health factors. In our model, “Smoking” means the agent smokes; “Not smoking” means the agent never smoked or quit smoking for more than one year. “Healthy diet” means the agent eats at least five fruits or vegetables per day; “Unhealthy diet” means the agent eats fewer than five fruits or vegetables per day. “Physically active” means the agent does more than 150 min of moderate physical activity per week; “Physically inactive” means the agent does fewer than 150 min of moderate physical activity per week; the behavioral factors may change at each time step, which is represented by the transition between “Smoking” and “Not smoking”, “Healthy diet” and “Unhealthy diet”, “Physically active” and “Physically inactive”.

“Normal weight” means the agent has a body mass index lower than 25 Kg/m^2^, while “Overweight” means the agent has a body mass index of more than 25 Kg/m^2^. We adopted this criterion of 25 as it was suggested by WHO (World Health Organization) [27]. “Without Hypertension” means the agent does not currently have hypertension; “Hypertension” means the agent has blood pressure of more than 140/90 mmHg. “Without Hypercholesterolemia” means the agent does not currently have hypercholesterolemia; “Hypercholesterolemia” means the agent has a cholesterol level of more than 230 mg/dL. “Without Nephropathy” means the agent has never been diagnosed with nephropathy; “Nephropathy” means the agent has been diagnosed with nephropathy. “No CVD history” means the agent has never been diagnosed with any cardiovascular disease; “MI history” and “Stroke history” means the agent has been diagnosed with myocardial infarction and stroke, respectively; “Death” means the agent lost his life. The factors of diabetic complications may change during the simulation time, which is represented by the transitions between different states (i.e., transitions between “Normal weight” and “Overweight”).

“Without diabetes” means the agent does not currently have diabetes; “Prediabetes” means the agent is in the prediabetic stage; “Diabetes” means the agent has developed diabetes. “Normal Retina” means the retina of this agent is healthy; “Diabetic Retina” means the agent has developed diabetic retina, and “Blindness” means the agent lost his sight. The factors of diabetes-related health outcomes may change during the simulation time, which is represented by the transitions between different states (i.e., transitions between “Without diabetes” and “Prediabetes”).

Some transitions are independent, while others are correlated. The transition probability summarized in Table 1 was estimated from the published literature. We adjusted the transition probabilities of behavior states with social influence; the adjusted probabilities are given by the following equation:

(1) $$p^{*}=p\cdot F,$$

where p is the original probability extracted from Table 1, $p^{*}$ is the adjusted probability, and F is the social influence on the behavioral factors. The calculation of F will be explained in the next section. 

### 3.2. The Person-to-Person Spread

The quantitative linkage between individual behavioral changes and the population health outcome is the key to understanding the diabetes epidemic. Network analysis is of great value to offering insight into understanding people’s behavioral changes [28]. All the agents in our model make up a social network, and we chose the Watts–Strogatz network structure, which was one of the most commonly used structures. The agents in the Watts–Strogatz network tend to form small social groups (known as a high clustering coefficient), and any individual is only a short social distance away from any other individual in the same network (known as small average distance) [29,30]. 

**Figure 2:**
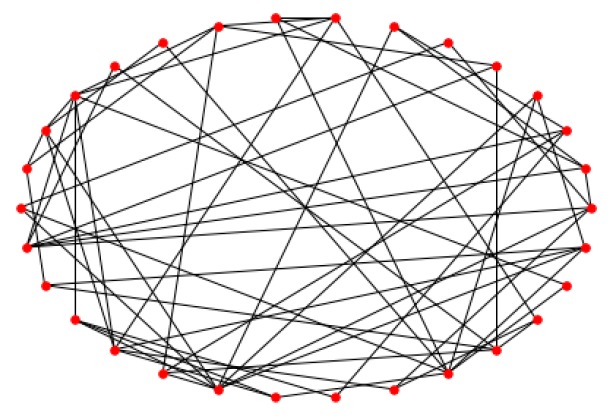
Ring lattice graph.

We generated 10,000 hypothetical individuals and formatted the social network. Our scenarios relied on a Watts–Strogatz network structure with a certain average degree (the average number of social ties per agent) and clustering coefficient (how closely the agents are connected).

a. We started by connecting all the agents into dense groups; we constructed a regular ring lattice (Figure 2), and; a graph with 10,000 nodes $N_{1},N_{2},\cdots N_{10,000}$ each connected to several neighbors on either side and in equal amounts. Each node represents one agent.

To describe the person-to-person spread of certain lifestyles quantitatively, we gave the agents a set of adaptive behavioral rules and defined how agents interact with each other.

b. Agents can affect others. For all agents $N_{1},N_{1},\cdots N_{10,000}$, agent $N_{i}$ can send positive and negative signals to his connected neighbors at the beginning of each simulation period, For example, If agent $N_{i}$ has healthy eating habits, he will send a positive signal. if agent $N_{i}$ has unhealthy eating habits, he will send a negative signal.
c. Agents can be affected by others. The agent who receives asignal, agent $N_{j}$ is likely to be affected by agent $N_{i}$’s positive or negative signal. For example, $N_{j}$ is more likely to keep or change to an unhealthy diet habit if he receives a negative signal, and he is more likely to keep or change to a healthy diet habit if he receives a positive signal.
d. The social influence is quantified. If an agent receives m positive signals and n negative signals, then his transition probability from “Unhealthy diet” to “Healthy diet” will be adjusted to $p^{*}=p\cdot F$, where p is the original transition probability extracted from Table 1 and F is the social influence factor; the value of social influence F is defined as $\frac{m}{n}$.
e. The transitions of smoking exercise habit are all the same; the more positive signals one agent receives, the more likely it is he will keep or change to a healthy smoking/exercising habit.

### 3.3. Population Health Outcome

Our model summarizes individual health progression and reports the population outcomes (diabetes-related behaviors, diabetic complications, and diabetes-related health outcome). In the following sections, we will focus on the population-level diabetes-related behavioral changes and diabetes rate. 

### 3.4. Model Parameters

The specifications of the model parameters were mainly estimated from the latest published paper and official datasets. It is worth emphasizing that the parameter estimation process required a large-scale literature review and synthesis to make the model useful.

The behavioral changes were independent of other health factors and affected by the social influence; we estimated the annual transition probability from “Not smoking” to “Smoking” based on the age-specific smoking cession survey of the WHO Global Health Observatory. The annual transition rate from “Unhealthy diet” to “Healthy diet” was estimated to be 0.03 for all ages [31], while the annual transition rate between “Physically active” and “Physically inactive” was estimated to be 0.049 for all ages [32]. The diabetes-related complications gradually progressed and affected each other. Body weight, for example, was closely related to daily diet habits and exercise habits [33]. The transition probability between “Normal weight” and “Overweight” was relative to the behavioral factors as well. We estimated the annual transition probabilities of diabetes-related complications and health outcomes based on a few published studies [33,34,35,36,37]. The specifications of transition probabilities and their correlations are summarized in Table 1.

### 3.5. Validation Procedure

The simulation approach is widely applied to solve public health problems so standard validation principles are well established [39]. The construction of the model, the conceptual model structure, the mathematic equations, and the coding part have been carefully checked by the authors, along with detailed consultations with medical experts, to guarantee the internal validity and face validity. To implement cross-validation, we compared our model to the models reviewed or built by the Roberts group [19,40]; among 22 diabetic modeling studies, 16 of them are Markov models. The conceptual model proposed in our paper is similar to these Markov models with two exceptions: (1) Our model is the first agent-based model applied to diabetic research. (2) Earlier models did not include the transition of diabetes-related behaviors and diabetic complications. We will present additional numerical results in the next section which demonstrate the predictive validity of our model.

## 4. Results

### 4.1. Predictive Validation Results

We generated a hypothetical population of 10,000 individuals based on the demographics and health profiles of general Chinese adults. The nationally representative data of China were extracted from the following datasets: The Chinese National Sample Survey, the WHO Global Health Observatory; the China Health and Nutrition Survey (CHNS) and data from Non-Communicable Diseases Risk Factor Collaboration (NCD Risc). The Chinese National Sample Survey is provided by the Chinese government and offers the official statistics about the demographic characteristics and health conditions of Chinese adults. The WHO Global Health Observatory provides health-related statistics for its member states, including China. The CHNS data is a questionnaire survey including indicators about nutrition, health behaviors, health conditions, and household and individual economic, demographic, and social factors. The NCD Risc Data is a dataset reporting the risk factors of non-communicable diseases published by the Non-Communicable Diseases Risk Factor Collaboration.

The demographics and health profiles of the initial population are summarized in Table 2. We simulated the population for five years without any intervention. Then, we compared the simulated results with the actual statistics estimated by the CHNS data for four factors (diabetes rate, overweight rate, smoking rate, and physically active rate). Our model showed a predictive power in the diabetes rate, overweight rate, and smoking rate, as shown in Table 3 presents, this proves the predictive validity of our model. Our model did not make reasonable prediction of the change of exercise habits meaning more accurate calibration is needed to improve our model.

### 4.2. Intervention Experiments

In this section, we will show how our model can be used in practice by assessing three hypothetical lifestyle interventions (“control smoking”, “promote healthy diet”, and “improve physical activity”). We simulated the population with different interventions for 5, 10, 15, and 20 years, separately. Specifically, “control smoking” means a lifestyle program implemented to reduce the proportion of the population who smokes by half, “promote healthy diet” means a lifestyle program implemented to reduce the proportion of the population who eats fewer than five fruits and vegetables per day by half, and “improve physical activity” means a lifestyle program implemented to reduce the proportion of the population who exercises fewer than 150 min per week by half. We also simulated the scenario without any interventions; and set this as the control group.

The results of the different lifestyle interventions are presented in Table 4. Compared to doing nothing, the three lifestyle interventions successfully reduced the population diabetes rate. “Improve activity” seems to have been the most effective intervention in preventing diabetes, followed by “promote healthy diet” and “control smoking”. Intervention experiments can also provide more precise insight to inform decision making. For example, the “improve activity” intervention reduced the population diabetes rate by 0.69% after 20 years, which means a reduction of 9.6 million diabetics (China has a population of 1.39 billion). This reduction can translate to a saving of approximately 4.464 billion dollars in a single year as the annual medical cost of a diabetic patient in China is about 465 dollars, according to the Chinese Diabetes Society [41]. The reduced proportion of diabetics after implementing the “promote healthy diet” intervention for 20 years means a saving of 4072 million dollars. The “control smoking” intervention yielded a reduction of 2.2 million diabetics and a saving of about 1.034 billion dollars. However, lifestyle intervention programs are not that costly. Publicizing healthy eating norms and exercise habits which lead people to adopt healthy lifestyles through media can be helpful and inexpensive. The Chinese government decreed a ban on smoking in public workplaces and on public transportation in 2011; this ban can greatly reduce the difficulty and cost to “control smoking”.

### 4.3. Sensitivity Analysis

Although lifestyle interventions have turned out to be effective in preventing diabetes, they also bring economic costs. Incontrovertibly, an intensive intervention is more costly than a normal intervention. Therefore, we implemented a sensitivity analysis toward the intensity level and compared the preventive effect of lifestyle interventions with different intensities. Each kind of intervention had the same five intensity levels, reducing the proportion of people who smoke by 10%, 20%, 30%, 40%, 50%, separately.

**Figure 3:**
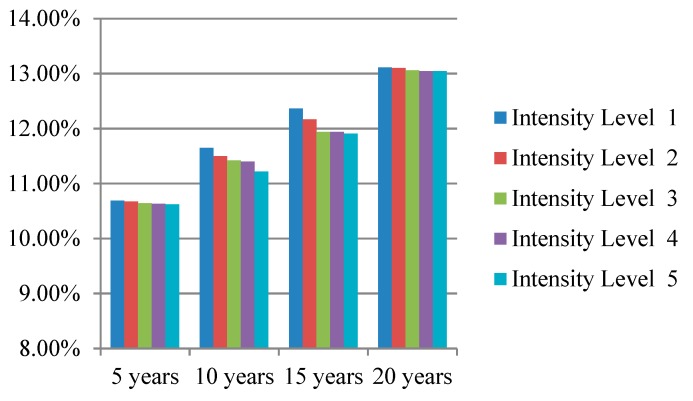
Diabetes rate after “control smoking” intervention with different intensity levels.

**Figure 4:**
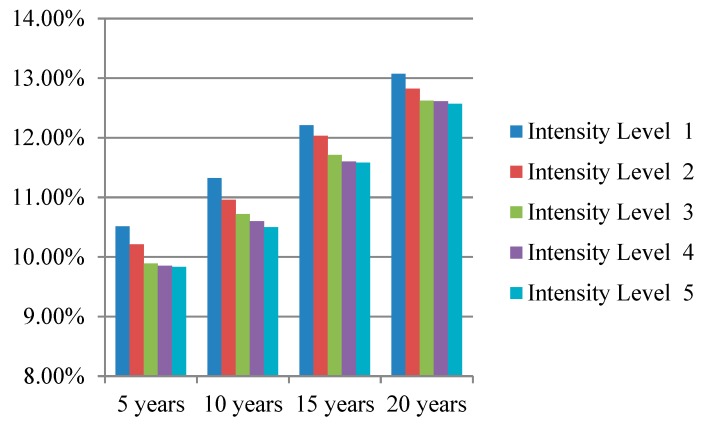
Diabetes rate after “promote healthy diet” intervention with different intensity levels.

**Figure 5:**
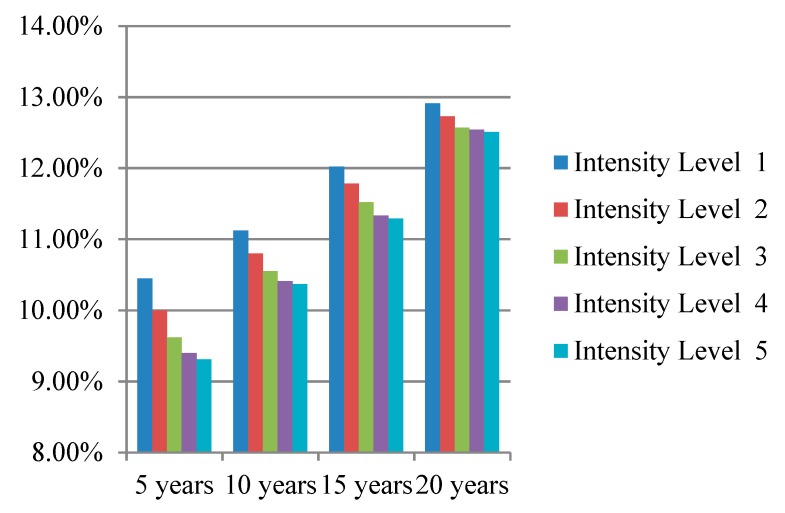
Diabetes rate after “improve activity” intervention with different intensity levels.

We simulated the prevention effectiveness of different lifestyle interventions with different intensity levels for 5, 10, 15, and 20 years; the simulation results are presented intuitively in Figure 3, Figure 4 and Figure 5. The preventive effect of interventions was quantitatively reflected in the population diabetes rate. “Improve activity” turned out to be the most effective intervention compared to “control smoking” and “promote healthy diet”. In most cases, higher invention intensity indicated a better preventive effect. Increasing the intensity of intervention consistently would not always result in equal significant improvement in prevention outcomes. It is worth noting that although the differences in prevention effectiveness of lifestyle interventions with different intensities was significant, the most dramatic impact happened at the beginning and then attenuated over time.

## 5. Discussion

This study developed an agent-based model to quantitatively describe the diabetes epidemic. We presented how an individual’s diabetic health condition progresses and how diabetes spreads from person to person intuitively. Then, we showed how the model can be used in practice by implementing some hypothetical experiments. Our study incorporated the network analysis approach and the chronic disease modeling method to provide precise insight to inform decision making. Compared to previous models which only focused on the progression of the disease [19] or just emphasized the spread pattern [16], we took a step forward to develop a comprehensive model to explain the diabetes epidemic. The model also provided a framework for modeling the epidemic of other diseases.

We chose China as our research region as it has the largest number of people with diabetes in the world. We calibrated the parameters of our model based on the nationally representative data. Considering the great financial burden brought by treating and preventing diabetes, public health policy makers face a big challenge in establishing the most cost-effective interventions without too many decision-making costs. Our study provides a simulation method to assess different interventions without spending resources on testing different intervention programs. The model can also be used in other countries and regions.

The population diabetes rates 5, 10, 15, and 20 years after implementing the “control smoking” intervention were 10.62%, 11.22%, 11.91%, and 13.04%, respectively; the population diabetes rate in 5, 10, 15, and 20 years after implementing the “promote healthy diet” intervention was 9.83%, 10.50%, 11.58%, and 12.57%, respectively; the population diabetes rate in 5, 10, 15, and 20 years after implementing the “improve activity” intervention were 9.31%, 10.37%, 11.29%, and 12.51%, respectively. The results indicate that “improve activity” is the most effective lifestyle intervention in preventing diabetes compared to “control smoking” and “promote healthy diet”. Physical inactivity is becoming the fourth leading risk factor for global mortality and a major risk factor for non-communicable diseases such as cardiovascular disease, diabetes, and certain types of cancer (World Health Organization, 2010). It is of great benefit to implement interventions targeting the improvement people’s exercise habits; the government can construct more public sports facilities and emphasize the importance of exercise through newspapers, television programs, and social media.

Surprisingly, the preventive effects of interventions did not always increase significantly as the intensity of the intervention increased. The sensitivity analysis of the intensity of the interventions indicated that the most dramatic impact happened at the beginning and then attenuated over time. One possible reason can be social influence: Social facilitation may accelerate behavioral changes and then magnify the difference in preventive effects of lifestyle interventions with different intensities, while social comparison may slow down the behavioral changes and then narrow the difference. Social influence can be leveraged when establishing a behavioral intervention policy to prevent diabetes.

This study has some limitations. More data are needed to train and calibrate our model, and as the hypothetical experiments we implemented were kept in an idealized form, more practical experiments can be designed in the future. This study only assessed behavioral interventions; taking pharmacologic interventions into account is also planned in our future research.

## 6. Conclusions

This study quantitatively described the diabetes epidemic using a modeling approach. We linked the individual-level behavior interaction and health condition with the population-level health outcome through a bottom-up agent-based model. Our modeling framework can also be applied to explore other problems such as potential epidemic and diabetic complications. We assessed the different intervention programs using a simulation without spending resources on implementing and testing them. Finally, the model we developed is a user-specific model, which means our model can be applied in different countries and cultures.

## Figures and Tables

**Table 1:** Model parameters.

Parameter Name	Parameter Value	Data Source
Smoking/No smoking transition	Age-specific	WHO Global Health Observatory
Healthy/unhealthy diet transition	Healthy-unhealthy: 0.03 Unhealthy-healthy: 0.03	Dalziel and Segal 2007 [31]
Physical active/inactive transition	Active-inactive: 0.049 Inactive-active: 0.049	Dalziel et.al. 2006 [32]
BMI status transition	Related to behavioral factors	Ogden et al. 2007 [34]; Kaukua et al. 2003 [35]; Pan et al. 2011 [33].
Hypertension state transition	Related to age and weight	Zhang et.al. 2013 [36]
Cholesterol state transition	Related to age and weight	Zhang et.al. 2013 [36]
Diabetes transition	Related to age and weight	Zhang et.al. 2013 [36]
Nephropathy transition	Without-with: 0.01; With-without: 0.0003	Heron et.al.2012 [37]
CVD transition	Age- and gender-specific	Anderson et al. 1991 [38]

**Table 2:** Initial population characteristics.

Parameter Name	Parameter Value	Data Source
Age distribution (including mean, std. var, min, max)	Correspondingly 43.23, 14.8, 20.0, 79.0	National Sample Survey 2006
Gender distribution	Female proportion 0.484	National Sample Survey 2006
HbA1c distribution	Mean 5.5, std. var 0.7, min3, max 11	Zhang et.al. 2013 [36]
No current smoking rate	0.753	WHO Global Health Observatory
Normal BMI rate	0.573	WHO Global Health Observatory
Physically active rate	0.762	WHO Global Health Observatory
Healthy diet rate	0.244	CHNS Data
No history of hypertension (proportion)	0.8087	NCD Risc data
No history of high cholesterol (proportion)	0.705	CHNS Data

**Table 3:** Comparison between simulated results and real data.

Measures	Year	CHNS Actual Data (%)	Simulated Results (%)	*p*-Value
**Diabetes rate**	2006	5.59	5.59	1.00
	2009	8.91	9.2	0.60
	2011	10.69	10.1	0.67
**Overweight rate**	2006	8.84	8.84	1.00
	2009	11.2	12	0.17
	2011	15.4	14.5	0.20
**Smoking rate**	2006	29.8	29.8	1.00
	2009	29.8	28.5	0.01
	2011	29.2	28.3	0.13
**Activity Physically active rate**	2006	68.8	68.8	1.00
	2009	68.4	62.1	<0.01
	2011	63	60.4	<0.01

**Table 4:** Diabetes rates after different lifestyle intervention programs.

Intervention	Time Interval (Years)	Diabetes Rate (%)
**Control Smoking**	5	10.62
	10	11.22
	15	11.91
	20	13.04
**Promote Healthy Diet**	5	9.83
	10	10.50
	15	11.58
	20	12.57
**Improve Activity**	5	9.31
	10	10.37
	15	11.29
	20	12.51
**Without Any interventions**	5	10.69
	10	11.72
	15	12.39
	20	13.20
